# Conceptualization of Risk Stratification Using Large Language Models to Predict Severe Mycoplasma pneumoniae Pneumonia

**DOI:** 10.7759/cureus.98251

**Published:** 2025-12-01

**Authors:** Adebanke Adeyemi, Swapan Nath

**Affiliations:** 1 College of Medicine, Anne Burnett Marion School of Medicine, Texas Christian University, Fort Worth, USA; 2 Medical Education and Clinical Microbiology, Anne Burnett Marion School of Medicine, Texas Christian University, Fort Worth, USA

**Keywords:** artificial intelligence and education, chatgpt, clinical reasoning skills, health-professions education, large language models, necrotizing pneumonia, predictive markers for severity

## Abstract

Background

Large language models (LLMs), such as OpenAI’s ChatGPT-4o3 and GPT-5 (Deep Research, Deep Thinking), have emerged as tools with reasonable efficacy in multiple domains of healthcare to enhance literature synthesis, support reasoning, and guide diagnostic and management framework development. In clinical domains, LLMs have been evaluated for readability, accuracy, and decision support, but their role in health professions education scholarship is still emerging. *Mycoplasma pneumoniae* pneumonia (MPP) is a self-limiting atypical pneumonia; however, a subset of cases progress to severe or necrotizing disease, even in otherwise healthy patients. Predicting such trajectories in immunocompetent adults remains difficult due to the variable nature of host susceptibility and immune factors. This study examines predictive features of severe MPP and explores how LLMs can be leveraged to construct a conceptual risk stratification framework for use in health professions, education scholarship, and research skills development.

Methodology

We analyzed a single case of necrotizing MPP in a 32-year-old woman to develop a conceptual risk-stratification framework. A structured literature review (2010-2024) was conducted to identify clinical, laboratory, and imaging predictors of severe or refractory MPP. These predictors were compared with the patient’s course and organized into a three-tier framework (low, moderate, high risk) using supervised LLM-assisted synthesis.

Results

The case demonstrated several high-risk predictors of severe MPP, including cavitary disease, a loculated exudative effusion, and pneumothorax. Mapping these findings against literature-derived predictors placed the patient in the high-risk tier of a three-level framework, illustrating how LLM-assisted synthesis can convert a single case into a structured, hypothesis-generating, conceptual risk stratification framework.

Conclusions

This framework illustrates how LLM-assisted synthesis can link complex case data with published predictors to support early recognition of severe MPP. It also highlights a replicable educational approach for transforming single-patient narratives into hypothesis-generating tools that foster AI literacy and research skills in health professions education. To ensure appropriate interpretive boundaries, we emphasize that the conclusions are theoretical and hypothesis-generating rather than empirically validated. Because alternative interpretations of the literature-derived predictors are possible and the model is grounded in a single case, the framework should not be construed as a generalizable risk tool. Instead, it represents an educational scaffold demonstrating how structured synthesis can support reasoning while acknowledging the need for future empirical evaluation across larger patient samples.

## Introduction

Large language models (LLMs) provide a novel approach to bridge case-based clinical narratives, literature synthesis, and conceptual model-building. Unlike traditional descriptive case reports, integration of LLMs enables systematic extraction of predictive markers from published literature and comparison with real-world patient data, advancing the clinician-AI partnership envisioned in recent scholarship [1,2]. Traditional case reports often stop at descriptive insights, limiting their impact on hypothesis generation or model development. In contrast, LLM-supported synthesis can extend single cases into structured, hypothesis-driven frameworks. We applied this approach to a case of necrotizing *Mycoplasma pneumoniae* pneumonia (MPP) to demonstrate how LLMs can objectively inform risk stratification and support educational scholarship in health professions education (HPE).

*M. pneumoniae* is a common cause of community-acquired pneumonia (CAP), typically producing a self-limited, mild illness in otherwise healthy individuals [3,4]. However, a small subset (0.5-2%) progresses to severe MPP, which may involve respiratory failure, diffuse consolidation, pleural effusions, or necrotizing changes [5-8]. Though rare, necrotizing pneumonia due to *M. pneumoniae* is occasionally reported, particularly in children and young adults, underscoring the need for early recognition of severe trajectories [6,9,10]. Because prediction remains difficult, innovative approaches, including AI-supported synthesis of predictors from the literature, may help extend insights from individual cases into conceptual frameworks useful for both clinical recognition and educational scholarship.

Predicting which patients will deteriorate from mild to severe disease remains challenging. Standard CAP scoring systems (e.g., CURB-65) are not well-calibrated for atypical pathogens or younger patients [11]. In *M. pneumoniae*, severity often reflects both pathogen load and dysregulated host immune response, including cytokine-driven tissue injury [4,10,11]. Reported clinical predictors of severe or refractory disease include prolonged fever, multilobar or cavitary infiltrates, elevated inflammatory markers (e.g., CRP, lactate dehydrogenase (LDH)), extrapulmonary involvement, and macrolide resistance [6-15].

In this report, we aim to construct a conceptual, rule-based risk-stratification framework for severe MMP by integrating case findings with literature-derived predictors using supervised LLM-assisted synthesis. The objective of this study was to examine literature-derived predictors of severe disease and demonstrate how supervised LLM-assisted synthesis can organize these predictors into a tiered model (low, moderate, high risk) aligned with the patient’s clinical trajectory. Our aim was to illustrate how a single case can be transformed into an educational, hypothesis-generating framework that supports early recognition of severe MPP and advances AI-integrated clinical reasoning in undergraduate medical education (UME).

The present paper illustrates how LLMs can be leveraged in case reports to conceptualize predictive frameworks for complex conditions, setting the stage for future validation studies and electronic health record (EHR)-integrated, multi-institutional risk-stratification modeling.

This work was previously presented in poster form at the Infectious Diseases Society of America Annual Meeting (IDWeek 2025; October 19-22, 2025; Atlanta, GA).

## Materials and methods

We report a case of necrotizing MPP in an immunocompetent adult. A 32-year-old previously healthy woman presented to the emergency department with a three-week history of progressively worsening respiratory symptoms. Her illness began with a headache, followed by fever and cough. She initially received a short course of azithromycin from urgent care but remained febrile, reaching temperatures up to 104°F (40°C). Subsequent treatment with amoxicillin and then doxycycline provided no improvement.

By day 12 of illness, chest imaging revealed pneumonia. Despite further outpatient therapy with cefpodoxime, her symptoms worsened, with persistent cough, pleuritic chest pain, shortness of breath, and development of dark brown sputum. A right-sided pleural effusion was noted. Chest CT demonstrated bilateral infiltrates with cavitary lesions, and thoracentesis confirmed an exudative effusion.

Her hospital course included empiric broad-spectrum antibiotics (meropenem and vancomycin), bronchoscopy, and chest tube placement. After nasopharyngeal PCR confirmed *M. pneumoniae*, therapy was narrowed to levofloxacin (for atypical pneumonia) and metronidazole (for any concomitant aspiration pneumonia and sustained clinical chronic course). Despite treatment, she developed cavitary necrosis, right apical pneumothorax, and persistent effusion. She had no comorbid conditions or evidence of immunodeficiency. The final diagnosis was necrotizing MPP complicated by exudative pleural effusion and pneumothorax.

LLM-augmented literature review

To contextualize the patient’s disease course, we conducted a structured literature review to identify clinical, laboratory, and imaging predictors of severe or refractory MPP. Searches were performed in PubMed and Embase for articles published between January 2010 and March 2024, with emphasis on studies from 2018 to 2024. Search terms included “severe *Mycoplasma pneumoniae* pneumonia,” “refractory *Mycoplasma pneumoniae* pneumonia,” “necrotizing,” “predictors,” and “risk factors.”

Inclusion criteria were peer-reviewed studies, systematic reviews, and meta-analyses that reported severity predictors in pediatric or adult MPP [5-10,12,16]. Exclusion criteria were non-English studies, single case reports without predictive analysis, and molecular-only studies lacking clinical correlation. Because of limited adult-specific data, pediatric findings were included, supported by literature suggesting consistent inflammatory and imaging markers across age groups [7,9,11].

These findings (N=214 records identified; N=63 full-text articles reviewed; N=14 retained for predictor extraction) served as the input for LLM-assisted synthesis, as described in the following section. To enhance replicability and address concerns about overreliance on software tools, we clarify that all steps in the literature review, predictor extraction, and framework construction followed a predefined, human-directed workflow. The LLM was used only as an assistive summarization tool, not as a data source. All searches, screening decisions, inclusion/exclusion judgments, and predictor classifications were performed manually by the authors. LLM outputs were used solely to organize text already derived from the retained peer-reviewed studies, and each suggested predictor was independently verified against the primary articles. No automated data-mining software, batch processing tools, or external databases outside PubMed/Embase were used. The screening process followed a systematic sequence involving an initial database search, removal of duplicates, full-text review, and final inclusion of eligible studies. The 14 retained studies together constituted the predictor extraction corpus, a curated collection of literature used to identify and extract predictive variables associated with specific outcomes. Non-MPP contextual references, including those related to AI, educational frameworks, or methodological approaches, were cited separately and were not included in this analytical flow.

Prompt engineering and output validation

To structure and expedite the literature synthesis, we used an LLM (ChatGPT-4o3/4.5/5 in Deep Research/Deep Thinking (DR/DT) modes). Prompts (Table 1) were iteratively refined to support stepwise reasoning and align with educational goals for hypothesis generation and conceptual model-building.

**Table 1:** Example Prompts for LLM-Augmented Literature Synthesis and Framework Development. Representative prompts were grouped into four categories: general extraction from literature (P1-P3), domain-specific predictor synthesis (P4-P6), case comparison and conceptual modeling (P7-P9), and refinement and review (P10-P11). Prompts were iteratively refined to support reproducibility, explainability, and educational value in the development of a conceptual risk stratification framework. LLM: large language model; CRP: C-reactive protein; LDH: lactate dehydrogenase

Prompt Number	Representative Prompts
P1	Identify peer-reviewed studies from the last 10 years that report predictors of severe or refractory *Mycoplasma pneumoniae* pneumonia (MPP) in children or adults.
P2	List commonly cited clinical, lab, and imaging features associated with severe or necrotizing MPP.
P3	Extract tiered predictors of severity in MPP from literature reviews or meta-analyses.
P4	What are the main clinical signs that predict a severe course of MPP?
P5	Summarize biomarkers that have been associated with disease severity in MPP, including leukocyte count, CRP, LDH, and others.
P6	Describe radiologic findings (e.g., infiltrates, effusion, cavitation) that correlate with severe disease in MPP.
P7	Given the following case features (summarized patient timeline), which severity predictors from literature are concordant with this case?
P8	Help group these predictors into three categories: clinical features, lab findings, and imaging.
P9	Based on these groupings, propose a three-tiered conceptual framework to stratify risk (low, moderate, high) in patients with suspected severe MPP.
P10	Critique the proposed framework based on limitations in literature (e.g., pediatric vs. adult, small sample sizes).
P11	How might the framework support medical education in undergraduate learners?

Prompts were deployed in consecutive MS3 student-led, mentor-supervised sessions to ensure transparency, reproducibility, and factual grounding. All LLM outputs were manually verified against PubMed-indexed sources to mitigate any “hallucination effect,” citation inaccuracies, or biased outputs. One of us, the supervising author (SKN), conducted iterative verification, consistent with New England Journal of Medicine (NEJM) AI recommendations for responsible LLM use in educational and conceptual research [1,2,17,18] and the author’s own published experience in prompt engineering in the Advancing AI Across Academic Medicine resource collection [19].

LLM sessions used ChatGPT-4o/5 (DR/DT modes). Temperature was kept low (0-0.2) for factual tasks and moderate (0.4) for synthesis. We disabled speculative generation and required citation placeholders in intermediate outputs to reduce any “hallucination effect.”

Prompt construction followed a structured approach: (i) defining the task (predictor extraction, case mapping, or framework generation), (ii) specifying required output format (lists, categories, or narrative synthesis), and (iii) embedding constraints (citation placeholders, avoidance of speculation, requirement to trace assertions to literature). Each prompt underwent iterative refinement across student-faculty sessions, during which outputs were evaluated for accuracy, relevance, and alignment with published data. Any discrepancies, overly general statements, or unverifiable claims were flagged and corrected through manual literature checks before inclusion in the predictor list or framework development.

Framework development

We designed this framework for UME as a structured approach through which learners apply three integrated domains - clinical features, laboratory indices, and imaging findings - to a de-identified case timeline. Learners assign a risk tier and justify their escalation decisions, thereby strengthening competencies in literature appraisal, model transparency, and bias or validation literacy. Findings from both the structured literature review and LLM-augmented synthesis were consolidated into a conceptual, rule-based risk stratification model. Within this framework, predictors encompassed prolonged fever, antibiotic non-response, and extrapulmonary manifestations as clinical indicators; leukocytosis and elevated inflammatory markers such as CRP and LDH as laboratory correlates; and multilobar involvement, cavitation, effusion, or pneumothorax as imaging determinants.

The patient’s case was subsequently mapped onto these domains to assess concordance. This process culminated in a three-tiered risk stratification framework (low, moderate, high) that demonstrates how case-derived data can guide both hypothesis generation and conceptual understanding of disease progression.

**Figure 1:**
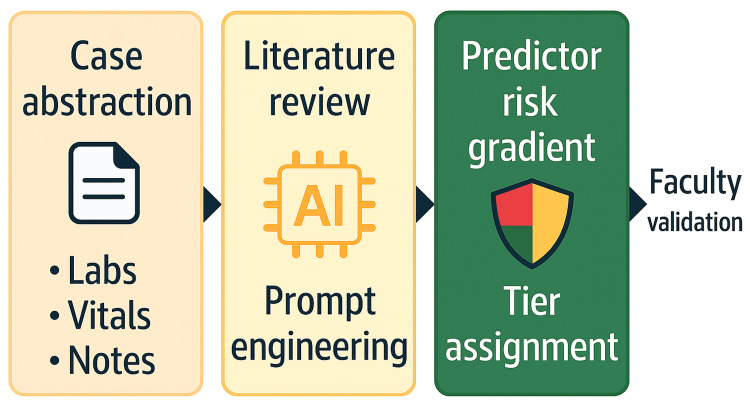
Workflow for LLM-Augmented Risk Stratification. The workflow involved a sequential process of case abstraction, literature review, prompt engineering/large language model (LLM) synthesis, followed by predictor risk-gradient assessment, risk-tier assignment, and faculty validation. The stratification step integrates predictor risk gradient → tier assignment, emphasizing supervised AI use and educational scaffolding rather than a validated clinical score. Image Credit: Generated using GPT-5 with human oversight; compliant with the Cureus AI image policy.

Figure 1 illustrates the workflow followed for the LLM-augmented risk-stratification framework using case data, literature review, prompt engineering, mapping of domains, risk tiering, and faculty validation.

Predictors were first classified according to a literature-derived risk gradient: high (e.g., cavitation, loculated effusion, pneumothorax, prolonged fever more than seven days despite macrolide), moderate (e.g., tachycardia, leukocytosis, extrapulmonary features), or low (e.g., LDH within the reference range of 140-280 U/L).

Tier assignment rules were then applied at the case level: High Tier ≥2 high-risk predictors, or any one high-risk predictor plus evidence of rapid progression; Moderate Tier = one high-risk predictor or ≥2 moderate-risk predictors; Low Tier = absence of the above. These tiering rules are conceptual and not validated.

## Results

Literature-derived predictive markers

The structured review identified multiple recurring predictors of severe or refractory MPP, which were organized into three primary domains: clinical, laboratory, and radiologic. Clinically, recurrent indicators of severe disease included prolonged fever despite macrolide therapy, persistent respiratory symptoms, and extrapulmonary manifestations such as rash, diarrhea, or neurologic findings, along with failure to improve after sequential antibiotic regimens [5-9,11]. Laboratory markers frequently associated with severity included serum LDH ≥480 U/L, leukocytosis or leukopenia, and elevated D-dimer or ferritin levels, particularly in patients with progressive or refractory courses [7,8,10,11,14]. Radiologic findings that correlated with severe disease included multilobar infiltrates, cavitation, and pleural effusions [6,8,10,12,16].

Additional risk indicators such as macrolide resistance, requirement for corticosteroids, and local complications like pneumothorax or empyema further strengthened the predictive matrix [7,11,16]. Collectively, these literature-derived predictors formed the reference framework against which the patient’s clinical trajectory was evaluated.

Case concordance/predictive marker comparison

The patient demonstrated alignment with several high-risk indicators. She had a persistent fever for over 12 days and failed three courses of antibiotics (azithromycin, doxycycline, cefpodoxime). Imaging revealed bilateral infiltrates with right lower lobe (RLL) cavitation measuring up to 2.5 cm. Thoracentesis confirmed a loculated, exudative pleural effusion, and a subsequent chest radiograph identified a right apical pneumothorax. Extrapulmonary features included rash and diarrhea. Laboratory findings showed leukocytosis (WBC 13.5 × 10^3^/µL).

Five high-risk markers were present: prolonged fever over seven days despite macrolide, macrolide and other antibiotics non-response (patient failed to respond to azithromycin, doxycycline, cefpodoxime); multilobar/cavitary disease; loculated/exudative pleural effusion; and pneumothorax. Three moderate-risk markers were also identified: tachycardia, leukocytosis, and extrapulmonary symptoms (rash and diarrhea).

Table 2 visually summarizes the literature-derived predictors alongside patient findings and risk gradient assignment.

**Table 2:** Predictors Versus Patient Findings in Necrotizing Mycoplasma pneumoniae Pneumonia. Patient features were mapped to literature-derived severity predictors/gradients for concordance, and tier assignment (high, moderate, low) was applied using pre-specified rules. WBC: white blood cell count; LDH: lactate dehydrogenase; RLL: right lower lobe; HR: heart rate

Predictor From Literature	Patient Finding	Reference Range	Risk Gradient	Concordance
Prolonged fever over seven days despite macrolide	Fever > 12 days prior to admission	-	High	Present
Tachycardia	HR 114 bpm at presentation	60-100 bpm	Moderate	Present
Leukocytosis	WBC 13.5 × 10^3^/µL	4.0-11.0 × 10^3^/µL	Moderate	Present
Serum LDH ≥ 480 U/L	Serum LDH 163 U/L	140-280 U/L	Low	Absent
Macrolide and other antibiotics non-response	Failed azithromycin, doxycycline, and cefpodoxime	-	High	Present
Multilobar/cavitary disease on CT	Bilateral infiltrates with a 2.5 cm cavitary lesion (RLL)	Normal: No cavitation or focal infiltrate	High	Present
Pleural effusion (loculated/exudative)	Loculated exudate: pleural protein 5.6 g/dL, LDH 465 U/L	Pleural protein < 3.0 g/dL; LDH < 200 U/L	High	Present
Pneumothorax	Right apical pneumothorax	Normal: Absent air in the pleural space	High	Present
Extrapulmonary features	Rash, diarrhea	-	Moderate	Present

In our patient, serum LDH was below the commonly cited severity threshold (163 U/L vs ≥480 U/L). Pleural LDH (465 U/L) confirmed the exudative nature of the effusion and contributed to the effusion risk domain. Bilateral infiltrates with a 2.5 cm cavitary lesion in the RLL were also noted.

Overall, the patient’s presentation aligned five high-risk and three moderate-risk features, placing her in the high-risk tier of the conceptual risk stratification framework. The strong concordance between this patient’s clinical course and published predictors supports her classification as high risk and illustrates how LLM-assisted synthesis can inform risk tiering in complex infectious disease presentations.

## Discussion

Here, we integrate clinical, laboratory, and radiologic predictors with the educational impact of leveraging LLMs to conceptualize a risk stratification framework as a form of HPE scholarship.

This case highlights necrotizing MPP in an otherwise healthy adult, an uncommon but fulminant manifestation of a typically self-limited infection. Several early warning signals pointed toward severity: fever persisting beyond one week despite macrolide therapy, failure of multiple antibiotic courses, extrapulmonary symptoms, and cavitary lung lesions on imaging [6,7]. Laboratory evaluation revealed leukocytosis, while serum LDH (163 U/L) remained below published severity thresholds (≥480 U/L). In contrast, pleural LDH (465 U/L) indicated an exudative effusion but did not independently contribute to systemic severity scoring [11]. Together, the findings of cavitary necrosis, pleural effusion, and pneumothorax delineated a high-risk trajectory consistent with severe disease.

By organizing these variables within a three-tiered predictor framework (low, moderate, and high risk), the case evolves from a descriptive clinical vignette into a structured, evidence-anchored framework of risk stratification. Clinically, this framework reinforces vigilance in any non-resolving pneumonia that fails standard therapy. Educationally, it models the cognitive integration that bridges bedside observation, laboratory data, imaging interpretation, and decision-making, enhancing learners’ diagnostic reasoning and situational awareness [3,5,9,14].

The novel contribution lies in the supervised use of LLMs (ChatGPT-4o3/4.5/5 DR, DT). The LLM supported literature synthesis and case mapping through three steps: (1) pattern recognition of predictors from peer-reviewed studies, (2) case concordance mapping, and (3) framework generation. This workflow transformed a single clinical case into a hypothesis-generating model. For medical students, the process-built skills in literature mining, risk factor synthesis, and framework design demonstrate how AI can scaffold scholarly output under faculty oversight [1,2].

This process illustrates how LLMs were explicitly leveraged to conceptualize a risk stratification framework as a form of UME scholarship. By embedding case features within a rule-based, literature-guided structure, the model demonstrates how single-patient narratives can be transformed into hypothesis-generating frameworks that advance both clinical reasoning and AI literacy for medical students.

Unlike opaque machine-learning models, the proposed framework relies on accessible data (fever, antibiotic response, imaging, biomarkers). Its transparency enhances reproducibility and educational use, though it is not a validated clinical tool. Instead, it serves as a starting scaffold for research, bedside teaching, and simulation-based training.

Machine learning approaches to severe MPP prediction have reported strong performance [8] but require complex datasets and external validation. Our rule-based framework offers complementary strengths: accessibility, interpretability, and direct integration into HPE. It provides a bridge between single-case scholarship and future data-driven model development [9,10].

This project illustrates how UME learners can progress from case → concept → model using AI-assisted synthesis. The process produced both a scholarly conference abstract and this paper, reinforcing its replicability for clerkship capstones and AI-integrated seminars. Such initiatives build competencies in AI literacy, bias awareness, and hypothesis-driven scholarship at the undergraduate level.

The limitation of this study is a single-case, conceptual report. Many predictors derive from pediatric studies, also limiting generalizability to adults [5,7,9,11]. Missing labs (e.g., CRP) restricted the completeness of scoring. LLM outputs remain dependent on training data quality and require human verification [1,2]. We explicitly note that while the workflow itself - literature search, manual screening, predictor extraction, and case-to-predictor mapping - can be replicated by other investigators, the precise LLM outputs cannot be reproduced verbatim due to the interpretive and conceptual nature of the model. All predictor lists, tiering rules, and framework structures were manually derived from the primary literature, with the LLM used only for assisted organization and summarization. No unverified or standalone AI-generated content was included. Future work applying the same methodology across larger patient samples may enable more formal validation and reproducibility of the tiering schema. Future studies should also validate the framework in larger adult cohorts, with outcomes such as ICU admission or radiographic progression. Hybrid approaches may combine rule-based transparency with machine learning scalability, subject to rigorous validation and bias auditing [17,18].

## Conclusions

A single case of severe or necrotizing MPP was reframed from descriptive narrative into a conceptual risk stratification framework through structured literature synthesis and supervised LLM support. By aligning published predictors with case features, the framework emphasizes early recognition of deterioration, transparent reasoning, and rule-based categorization. This illustrates how LLMs can be leveraged to conceptualize risk stratification frameworks as a form of HPE scholarship, offering a repeatable scaffold for student learning, simulation-based training, and future hypothesis-driven validation.
